# Prime Editing of Phytoene Synthase 1 in Rice for Seed Carotenoid Biofortification

**DOI:** 10.1111/pbi.70697

**Published:** 2026-06-09

**Authors:** Byeong‐Hoon Kim, Yeo Jin Lee, Yong Jin Choi, Sooyeon Lim, Young Jin Park, Jae Kwang Kim, Sun‐Hwa Ha

**Affiliations:** ^1^ Graduate School of Green‐Bio Science, College of Life Sciences Kyung Hee University Yongin Korea; ^2^ Division of Life Sciences and Bioengineering Incheon National University Incheon Korea

**Keywords:** Golden Rice, prime editing, transgene‐free

Carotenoids are vital for human health as provitamin A sources and antioxidants, yet their levels remain low in staple crops. The development of ‘Golden Rice’ set a milestone in carotenoid biofortification through endosperm‐specific expression of *PSY* and *CrtI*, with subsequent efforts focusing on PSY due to its rate‐limiting role in carotenoid biosynthesis (Giuliano 2017; Zhou et al. 2022). However, CRISPR‐Cas‐based gain‐of‐function strategies targeting PSY remain limited in cereal crops. Here, we evaluated natural PSY variants for enhanced enzymatic activity and introduced a mutation into the *OsPSY1* locus via prime editing, resulting in increased seed carotenoid accumulation with Golden Rice‐like traits.

Previous genome editing efforts for gain‐of‐function modification of *PSY* include adenine base editing of *ClPSY1* introducing the K149E allele in watermelon, the suggested to affect substrate‐binding affinity (Tian et al. 2026). To enhance PSY activity via genome editing, we selected two natural variants and one artificial mutant previously associated with increased carotenoid biosynthesis: MePSY2^A191D^ from a yellow‐rooted cassava, ZmPSY1^P257T^ from predominantly yellow endosperm maize, and SlPSY1^N136Y^ from active tomato SlPSY2 (Welsch et al. 2010; Shumskaya et al. 2012; Cao et al. 2019). Finally, A184D and P267T were selected as candidate golden SNPs (Figure S1a), following protein sequence alignment that identified corresponding the residues in rice as OsPSY1^A184^, OsPSY1^P267^, and OsPSY1^Y144^ (Figure S1b).

To assess their effects on carotenoid biosynthesis, three *OsPSY1* variants—*OsPSY1* ^A184D^, *OsPSY1* ^P267T^, and *OsPSY1* ^A184D/P267T^—were generated by site‐directed mutagenesis based on *OsPSY1* ^WT^ (Table S1). After removing the transit peptide (TP) predicted by UniProt (Table S2), *E. coli* harbouring *pAC‐85b* (lacking *CrtB*) was transformed with each truncated variant, with A184D‐containing variants showing increased β‐carotene production (~1.3‐fold) and enhanced yellow pigmentation compared to the wild‐type and P267T variant; this trend was recapitulated in rice calli expressing full‐length variants, which exhibited ~4‐fold higher β‐carotene levels, *de novo* α‐carotene production, and distinct orange coloration (Figure 1a). Positioned near the Mg2+ binding D‐rich motif (Figure S1), the A184D substitution did not alter the overall 3D structures of OsPSY1 but increased polar contacts with T181 from two to four (Figure 1b). Molecular dynamics simulations further indicated enhanced local stability under substrate‐free conditions and stabilization of the active‐site lid in the presence of GGPP, as evidenced by reduced RMSF (Figure S2b,d). Consistently, in vitro kinetic assays based on PPi release revealed ~5‐fold higher substrate affinity and increased catalytic efficiency (Figure S3). Together, these findings demonstrate that the cassava‐derived A184D substitution markedly enhances OsPSY1 activity.

**FIGURE 1:**
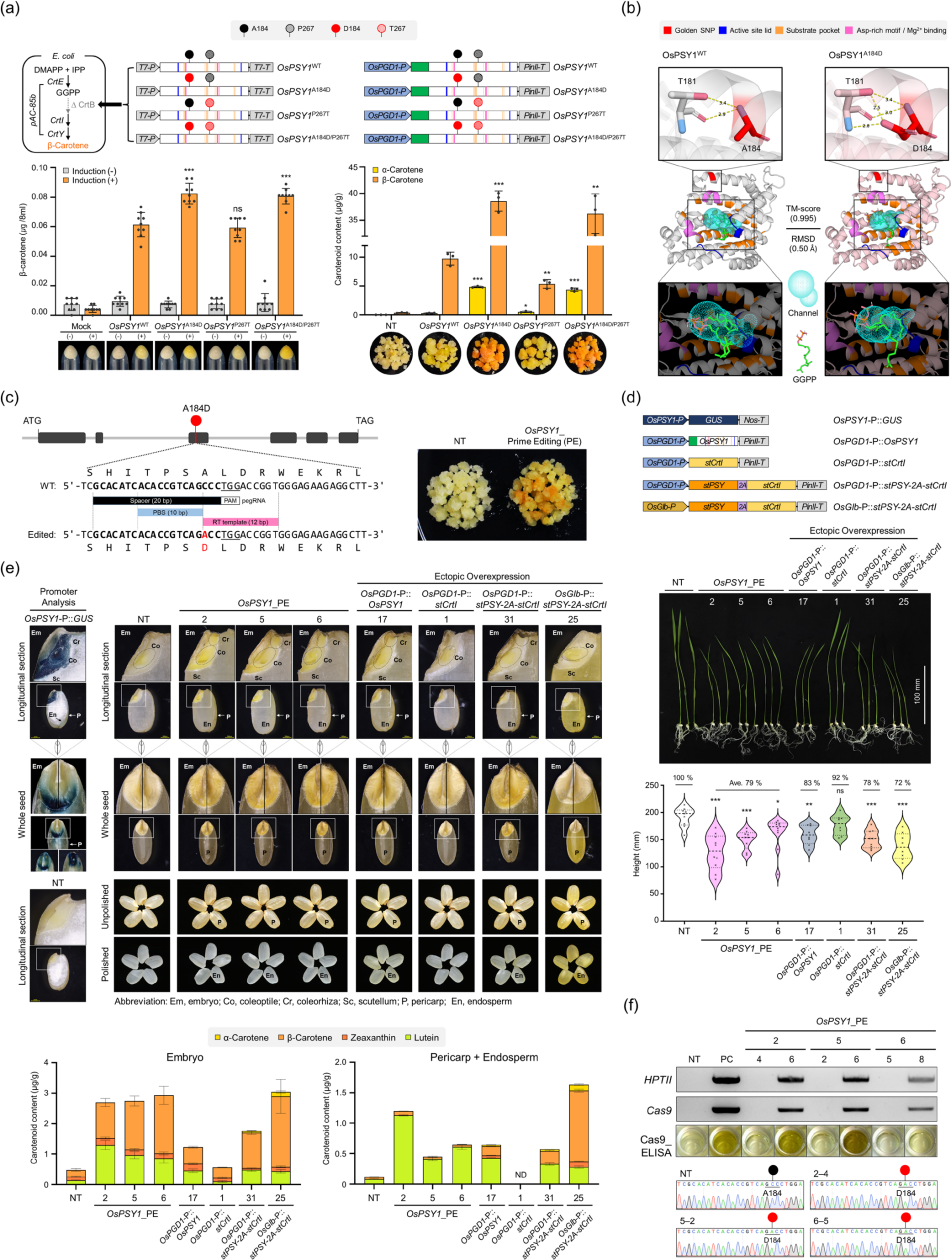
Prime editing–mediated engineering of *OsPSY1* enhances carotenoid accumulation in rice seeds. (a) Functional analysis of *OsPSY1* variants in *E. coli* and rice calli, showing enhanced carotenoid production and accumulation. (b) Structural modelling of OsPSY1 variants with substrate docking, highlighting changes in the local environment and active‐site configuration. (c) pegRNA design for *OsPSY1* prime editing, with representative colour phenotypes of prime‐edited rice calli prior to regeneration. (d) Schematic of overexpression constructs and seedling phenotypes at 9 days after germination with plant height quantification (*n* = 10). (e) In seeds, tissue‐specific promoter activity (GUS), carotenoid‐derived pigmentation, and carotenoid content and composition quantified by HPLC (mean ± SD, *n* = 3). ND, not detected. (f) Validation of transgene‐free prime‐edited lines by PCR, Cas9 ELISA, and Sanger sequencing.

To introduce the A184D allele into the endogenous *OsPSY1* locus, we used prime editing (PE) to install a single C‐to‐A substitution using an enhanced plant PE2 system (Li et al. 2022). A pegRNA comprising a spacer, PAM, primer binding site (PBS), and reverse transcriptase (RT) template was designed, and orange coloration was observed in calli during transgenic selection; all regenerated *OsPSY1*_PE lines carried the intended edit, indicating 100% efficiency (19/19 lines; Figure 1c). To compare their plant phenotypes, we generated one promoter analysis construct (*OsPSY1*‐P::*GUS*) and three constitutive overexpression constructs (*OsPGD1*‐P::*OsPSY1*, *OsPGD1*‐P::*stCrtI*, and *OsPGD1‐*P::*stPSY‐2A‐stCrtI*, where *st* denotes a rice codon‐optimized synthetic gene), producing comparable numbers of transgenic lines (Figure 1d). Together with previously reported *OsGlb*‐P::*stPSY‐2A‐stCrtI* lines (Ha et al. 2019), transgene integration and copy number were confirmed (Figure S4a,b). Phenotypic analysis showed that seedlings expressing *OsPSY1*_PE, *OsPGD1*‐P::*OsPSY1*, or *OsPGD1*‐P::*stPSY‐2A‐stCrtI* exhibited similarly reduced plant height compared with *OsPGD1*‐P::*stCrtI* and non‐transgenic (NT) controls (Figure 1d; Figure S4c), indicating that *OsPSY1* prime editing phenocopies ectopic overexpression of *PSY* genes (*OsPSY1* or *stPSY*), likely by redirecting metabolic flux toward carotenoids at the expense of gibberellin biosynthesis.

To predict the spatial pattern of *OsPSY1*_PE‐driven pigmentation in seeds, a 3065 bp *OsPSY1* promoter was used to drive GUS expression, revealing strong GUS signals in embryo tissues (coleoptile, coleorhiza, and scutellum) and the pericarp (Figure 1e). Consistent with this pattern, *OsPSY1*_PE lines exhibited strong golden coloration in the embryo tissues and the pericarp, resembling constitutive *PSY* overexpression lines (*OsPGD1*‐P::*OsPSY1* and *OsPGD1‐*P::*stPSY‐2A‐stCrtI*), whereas NT and *OsPGD1*‐P::*stCrtI* showed faint pigmentation and *OsGlb*‐P::*stPSY‐2A‐stCrtI* displayed endosperm‐dominant coloration (Figure 1e; Figure S5). Meanwhile, pigmentation in the endosperm *OsPSY1*_PE lines was weaker than that of *OsPSY1* overexpression lines, likely reflecting lower expression driven by the endogenous *OsPSY1* promoter compared to the *OsPGD1* promoter (Figure S6). HPLC analysis quantitatively supported these observations (Figure 1e; Table S3). Compared with an *OsPGD1*‐P::*OsPSY1* line 17 carrying a single–copy insertion, *OsPSY1*_PE line 2 showed a 2.2‐fold increase in embryos and a 1.9‐fold increase in non‐embryo tissues (pericarp + endosperm). Embryo carotenoid levels reached 93% of those in seed‐specific *stPSY‐2A‐stCrtI* line 25, with non‐embryo tissues reaching up to 78%. These results highlight the potential of *OsPSY1* prime editing to achieve Golden Rice–like carotenoid accumulation.

By introducing the cassava‐derived A184D SNP into the *OsPSY1* locus via prime editing, we achieved carotenoid biofortification comparable to overexpression. Transgene‐free edited lines were isolated, avoiding GMO‐related regulatory constraints (Figure 1f; Figure S7). While endosperm expression from the endogenous *OsPSY1* promoter remains modest and carotenoid flux is biased toward lutein, these limitations highlight opportunities for further optimization. Strengthening and refining *OsPSY1* expression through promoter engineering, together with modification of branch‐point enzymes to favour β‐carotene accumulation, represents a coordinated strategy for further improvement. Collectively, these approaches provide a framework for developing carotenoid‐enriched, foreign‐DNA‐free cereal crops with direct relevance to human health.

## Author Contributions

B.‐H.K. performed experiments; Y.J.L. generated transgenic rice; Y.J.C. conducted structural modelling; S.L. assayed enzyme activity; Y.J.P. and J.K.K. analysed carotenoids; S.‐H.H. conceived and supervised the study and wrote the manuscript with B.‐H.K; all approved.

## Funding

This work was supported by the Rural Development Administration, Republic of Korea, RS‐2024‐00322447; National Research Foundation (NRF) of Korea, RS‐2024‐00440478, RS‐2024‐00347806, RS‐2024‐00407469.

## Conflicts of Interest

The authors declare no conflicts of interest.

## Supporting information


**Figure S1–S7** and **Table S1–S3**.

## Data Availability

The data that support the findings of this study are available in Supporting Information.
