# Supplementary material for: Prime Editing of Phytoene Synthase 1 in Rice for Seed Carotenoid Biofortification
Source: Plant Biotechnol J. 2026 Jun 9:10.1111/pbi.70697. Online ahead of print. doi: 10.1111/pbi.70697 (PMC13398656; doi:10.1111/pbi.70697)
Supplement: Supplementary file 1 — Figure S1–S7 and Table S1–S3. [file PBI-9999-0-s001.docx]

**Supporting Information**

**Prime Editing of Phytoene Synthase 1 in Rice for Seed Carotenoid Biofortification**

Byeong-Hoon Kim^1^, Yeo Jin Lee^1^, Yong Jin Choi^1^, Sooyeon Lim^1^, Young Jin Park^2^, Jae Kwang Kim^2^, and Sun-Hwa Ha^1^ *

**Materials & Methods**

**Cloning and generation of *OsPSY1* variants by site-directed mutagenesis**

A 1,537 bp *OsPSY1* cDNA (1,263 bp CDS) was amplified from total RNAs of rice seedlings. *OsPSY1* variants carrying single- or double-point mutations—C551A (A184D), C799A (P267T), and C551A/C799A (A184D/P267T)—were generated by overlap-extension PCR using mutation-specific primer pairs (Table S3).

**Activity assessment of *OsPSY1* variants in *E. coli***

TP-truncated *OsPSY1* variants were cloned into *pET28a(+)* using primer sets (Table S3) and co-transformed into *E. coli* harboring the *pAC-85b* plasmid, which provides *CrtE*, *CrtI*, and *CrtY* for β-carotene production but lacks the *CrtB* encoding bacterial PSY (Cunningham and Gantt 2007). Following overnight induction with IPTG and arabinose at 0.5 OD_600_, bacterial pellets were visually assessed and used to extract carotenoid using 80% (v/v) acetone with sonication. Total carotenoid content was quantified spectrophotometrically at 470 nm using established equations (Wellburn 1994).

**Transient overexpression of *OsPSY* variants in rice calli**

*OsPSY1* variants were cloned into the binary vector *pPZP200_OsPGD1*-P*_3′PinII* under the control of the constitutive *OsPGD1* promoter (Park et al. 2010). Rice calli were induced from mature seeds of *Japonica*-type Korean rice (*Oryza sativa* L. cv. Dongjin) on N6 medium for 10 days. Each construct was introduced into *Agrobacterium tumefaciens* and then used to infect rice calli, followed by 3 days of co-cultivation. After washing, calli were transferred to selection medium and cultured in darkness for 10 weeks. Callus pigmentation was visually assessed and carotenoids were quantified by HPLC following a previously described method (Lee et al. 2024).

**Computational analysis of protein structure and molecular dynamics**

Putative TP and transmembrane (TM) regions of OsPSY1 were predicted using online algorithms: UniProt (https://www.uniprot.org/ Q5Z5B7), TMHMM (https://services.healthtech.dtu.dk/services/TMHMM-2.0/), DAS (https://tmdas.bioinfo.se/), and MemBrain 3.1 (https://www.csbio.sjtu.edu.cn/bioinf/MemBrain/). The 3D structures of TP-truncated proteins were predicted using AlphaFold3 (Abramson et al. 2024). Ligand-binding pockets and channels were analyzed using PrankWeb (Jendele et al. 2019) and MOLEonline server (Pravda et al., 2018). Structural alignment and root-mean-square deviation (RMSD) calculations were performed using TM-align and UCSF ChimeraX (Zhang and Skolnick 2005; Goddard et al. 2018), with visualization in PyMOL (v3.0). Molecular dynamics (MD) simulations were conducted using GROMACS (Abraham et al. 2015) for both apo and GGPP-bound models, with docking performed using AutoDock Vina (Eberhardt et al. 2021). Systems were energy-minimized and equilibrated under NVT and NPT conditions using standard protocols. System stability and flexibility were assessed by RMSD and Cα root- mean-square fluctuation (RMSF), respectively.

**Purification of recombinant OsPSY1 variants and PSY activity assay**

TP-truncated OsPSY1 variants were expressed as N-terminal maltose-binding protein (MBP) fusion in *E. coli* Rosetta2(DE3)pLysS cells using *pMAL* vector system (Novagen). Following induction with 0.1 mM IPTG at 15 °C for 24 h, cells were lysed by sonication, and proteins were purified using amylose affinity chromatography (New England Biolabs). Protein purity and concentration were verified by both SDS–PAGE and Bradford assay (Bio-Rad). Enzymatic activity was measured by quantifying inorganic pyrophosphate (PPi) release during GGPP-to-phytoene conversion (Rodríguez-Concepción and Welsch 2020). Reactions were performed at 25 °C in buffer containing Tris–HCl, MgCl₂, MESG, purine nucleoside phosphorylase, and inorganic pyrophosphatase, with GGPP concentrations ranging from 2.5 to 50 µM. Each reaction contained 5 µg of purified protein. Kinetic parameters (*K_m_* and *k_cat_*) were determined by fitting initial velocities to the Michaelis–Menten equation.

**Binary vector construction and stable rice transformation**

For prime editing, two pegRNAs targeting *OsPSY1* locus were designed via RGEN Tools (http://www.rgenome.net) and introduced into the *pPEmax* backbone, which employs a composite U6 promoter (CaMV35S-CmYLCV-U6) and an evopreQ1 motif to enhance pegRNA stability (Li et al., 2022). For promoter analysis, a 3,065 bp *OsPSY1* promoter region (including the 565 bp 5′ UTR) was cloned into *pCAMBIA1305.1*, to drive GUS expression. For overexpression, *OsPSY1* and *stCrtI* were individually cloned into the *pPZP200_OsPGD1-*P*_3ZP200_*, while the *stPSY–2A–stCrtI* (formerly *stPAC*), comprising rice codon-optimized synthetic (*st*) versions of *PSY* and *CrtI* linked by a 2A peptide and previously used for endosperm-specific expression in Korean Golden Rice (Jeong et al. 2017), was cloned into the *pPZP600*_*OsPGD1*-P_*3′PinII*. Primer sequences are listed in Table S3. *Agrobacterium*-mediated transformation and plant regeneration were performed using embryogenic calli derived from mature seeds of *Japonica* rice (*Oryza sativa* L. cv. Dongjin) following established protocols (Lee et al. 2024).

**Molecular characterization and phenotypic analysis of transgenic plants**

Genomic DNA was extracted from rice leaves using the NucleoSpin Plant II kit (Macherey-Nagel). Homozygous T_0_ prime-edited lines were identified by Sanger sequencing of the *OsPSY1* locus. In T_0_ overexpression lines, T-DNA integration was confirmed by genomic PCR using gene-specific primers (Table S3), and copy number was determined by TaqMan qPCR targeting the *Nos* terminator, with the rice α-tubulin gene (Os11g14220) as an internal reference (Lee et al. 2024). For histochemical GUS assays, seeds were vacuum-infiltrated with staining solution, incubated at 37 °C overnight, and cleared with 95% ethanol prior to imaging, following a previously described leaf protocol with minor modifications (Choi et al. 2025). For seedling phenotypes, seeds were germinated on 1/2 MS medium at 28 °C under a 16-h light/8-h dark photoperiod, seedling height was measured at 9 days after germination (*n* = 10 per line).

***In silico* gene expression analysis**

Spatiotemporal transcriptome data were obtained from the Rice Online Analysis Database v2 (ROADv2; Hwang et al. 2024). Expression profiles of *OsPSY1* and *OsPGD1* were extracted and visualized as heatmaps using GraphPad Prism. Relative expression levels were represented as color gradients, with schematic diagrams summarizing tissue-specific patterns.

**Seed color phenotyping and carotenoid analysis**

Mature seeds (60 days after flowering) were air-dried for 30 days, stored at 18 °C, and used for subsequent analyses as dehusked unpolished and polished seeds (TR-200 Rice Husker and Pearlest Polisher; Kett, Tokyo, Japan). For tissue-level color phenotyping, dry seeds were longitudinally sectioned using a razor blade, and both intact and sectioned seeds were imaged using a stereomicroscope (Olympus SZX16). For tissue-specific carotenoid analysis, seeds were dissected into embryo and the non-embryo fractions (pericarp + endosperm), and carotenoids were quantified in embryos, non-embryo fractions, and whole grains by HPLC following a previously described method (Lee et al. 2024).

**Selection of T-DNA–free lines**

To confirm the absence of vector-derived sequences in prime-edited rice lines, genomic PCR was performed targeting the *HPTII* and *Cas9* coding regions (Table S3). For protein-level validation, total proteins were extracted using a buffer containing Tris–HCl (pH 7.5), glycerol, NaCl, MgCl₂, EDTA, and protease inhibitors, and Cas9 protein was detected using a Cas9 ELISA Kit (GenScript) according to the manufacturer’s instructions, with absorbance measured at 450 nm using a microplate reader. In addition, edits at the *OsPSY1* locus were confirmed by Sanger sequencing.

**Supplementary References**

Abraham, M. J., Murtola, T., Schulz, R., et al. 2015. "GROMACS: High Performance Molecular Simulations through Multi-Level Parallelism from Laptops to Supercomputers."*SoftwareX***1:** 19–25.

Abramson, J., Adler, J., Dunger, J., et al. 2024. "Accurate Structure Prediction of Biomolecular Interactions with AlphaFold 3."*Nature***630:** 493–500.

Choi, H., Yi, T.G., Gho, Y., et al. 2025. "Editing of Rice PSEUDO-ETIOLATION IN LIGHT microProtein Genes Promotes Chloroplast Development."*The Plant Cell***37:** koaf235.

Cunningham Jr, F. X. and Gantt, E. 2007. "A Portfolio of Plasmids for Identification and Analysis of Carotenoid Pathway Enzymes: Adonis Aestivalis as a Case Study."*Photosynthesis Research***92:** 245–259.

Eberhardt, J., Santos-Martins, D., Tillack, A.F. and Forli, S. 2021. "AutoDock Vina 1.2. 0: New Docking Methods, Expanded Force Field, and Python Bindings."*Journal of Chemical Information and Modeling***61:** 3891–3898.

Goddard, T. D., Huang, C.C., Meng, E.C., et al. 2018. "UCSF ChimeraX: Meeting Modern Challenges in Visualization and Analysis."*Protein Science***27:** 14–25.

Hwang, W., Hong, W., Kim, E., et al. 2024. "The Rice Online Expression Profiles Array Database Version 2 (ROADv2): An Interactive Atlas for Rice Functional Genomics."*Rice***17:** 75.

Jendele, L., Krivak, R., Skoda, P., et al. 2019. "PrankWeb: A Web Server for Ligand Binding Site Prediction and Visualization."*Nucleic Acids Research***47:** W345–W349.

Jeong, Y. S., Ku, H., Kim, J.K., et al. 2017. "Effect of Codon Optimization on the Enhancement of the Β-Carotene Contents in Rice Endosperm."*Plant Biotechnology Reports***11:** 171–179.

Lee, Y. J., Jung, Y.J., Kim, J.H., et al. 2024. "Molecular Protocol to Develop Β-Carotene-Biofortified Rice Events Via Molecular Optimization."*Plant Physiology and Biochemistry***215:** 109051.

Li, J., Chen, L., Liang, J., et al. 2022. "Development of a Highly Efficient Prime Editor 2 System in Plants."*Genome Biology***23:** 161.

Park, S., Yi, N., Kim, Y.S., et al. 2010. "Analysis of Five Novel Putative Constitutive Gene Promoters in Transgenic Rice Plants."*Journal of Experimental Botany***61:** 2459–2467.

Pravda, L., Sehnal, D., Toušek, D., et al. 2018. "MOLEonline: A Web-Based Tool for Analyzing Channels, Tunnels and Pores (2018 Update)."*Nucleic Acids Research***46:** W368–W373.

Rodríguez-Concepción, M. and Welsch, R. 2020. "A Simple In Vitro Assay to Measure the Activity of Geranylgeranyl Diphosphate Synthase and Other Short-Chain Prenyltransferases." *Methods in Molecular Biology* **2083:** 27-38.

Wellburn, A. R. 1994. "The Spectral Determination of Chlorophylls a and B, as Well as Total Carotenoids, using various Solvents with Spectrophotometers of Different Resolution."*Journal of Plant Physiology***144:** 307–313.

Zhang, Y. and Skolnick, J. 2005. "TM-Align: A Protein Structure Alignment Algorithm Based on the TM-Score."*Nucleic Acids Research***33:** 2302–2309.


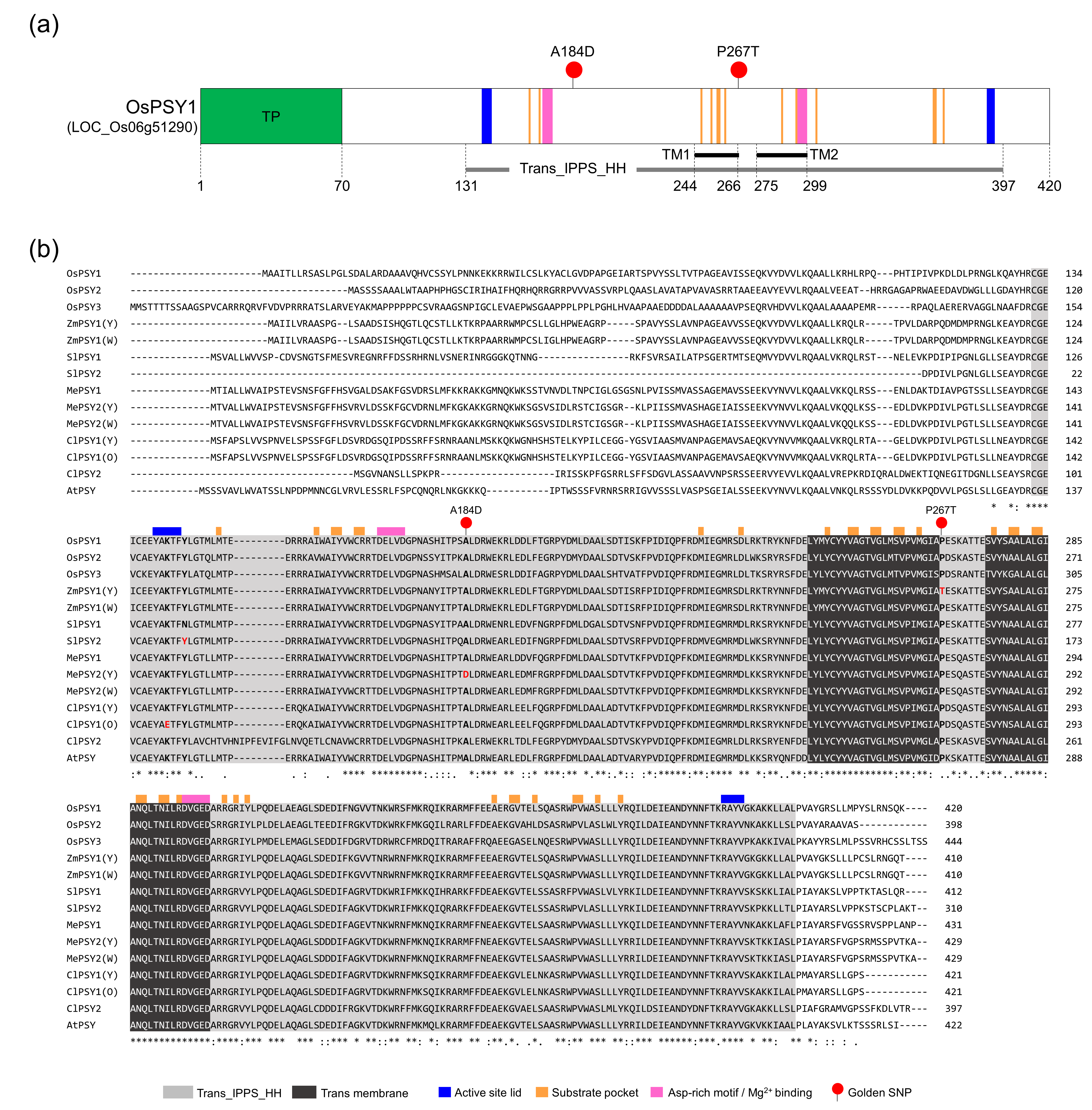


**Figure S1. Protein domain organization and sequence features of OsPSY1** (a) Schematic of the OsPSY1 protein indicating the N-terminal transit peptide (TP) and selected amino acid substitutions. (b) Protein sequence alignment among plant PSY orthologs with the following sequences (accession numbers in parentheses): AtPSY (AAA32836), ClPSY1 (ABC75827), ClPSY2 (Cla005425), MePSY1 (ACY42664), MePSY2 (W; ACY42669), MePSY2 (Y; ACY42670), OsPSY1 (CAG29391), OsPSY2 (BAG93381), OsPSY3 (ACI62767), SlPSY1 (AAA34153), SlPSY2 (AAA34187), ZmPSY1 (AAX13806), ZmPSY2 (AAX13807), and ZmPSY3 (ACG30201). Distinct color phenotypes are indicated by abbreviations in parentheses after each taxon: Y (yellow), W (white), and O (orange). Red letters within the alignment denote residues reported in previous studies to confer higher carotenoid accumulation in literatures. Annotated features include the trans-isoprenyl diphosphate synthase homology domain (Trans_IPPS_HH; head-to-head–type), transmembrane regions, the active site lid, substrate pocket residues, the Asp-rich motif/ Mg^2+^ binding site, and Golden SNPs. **
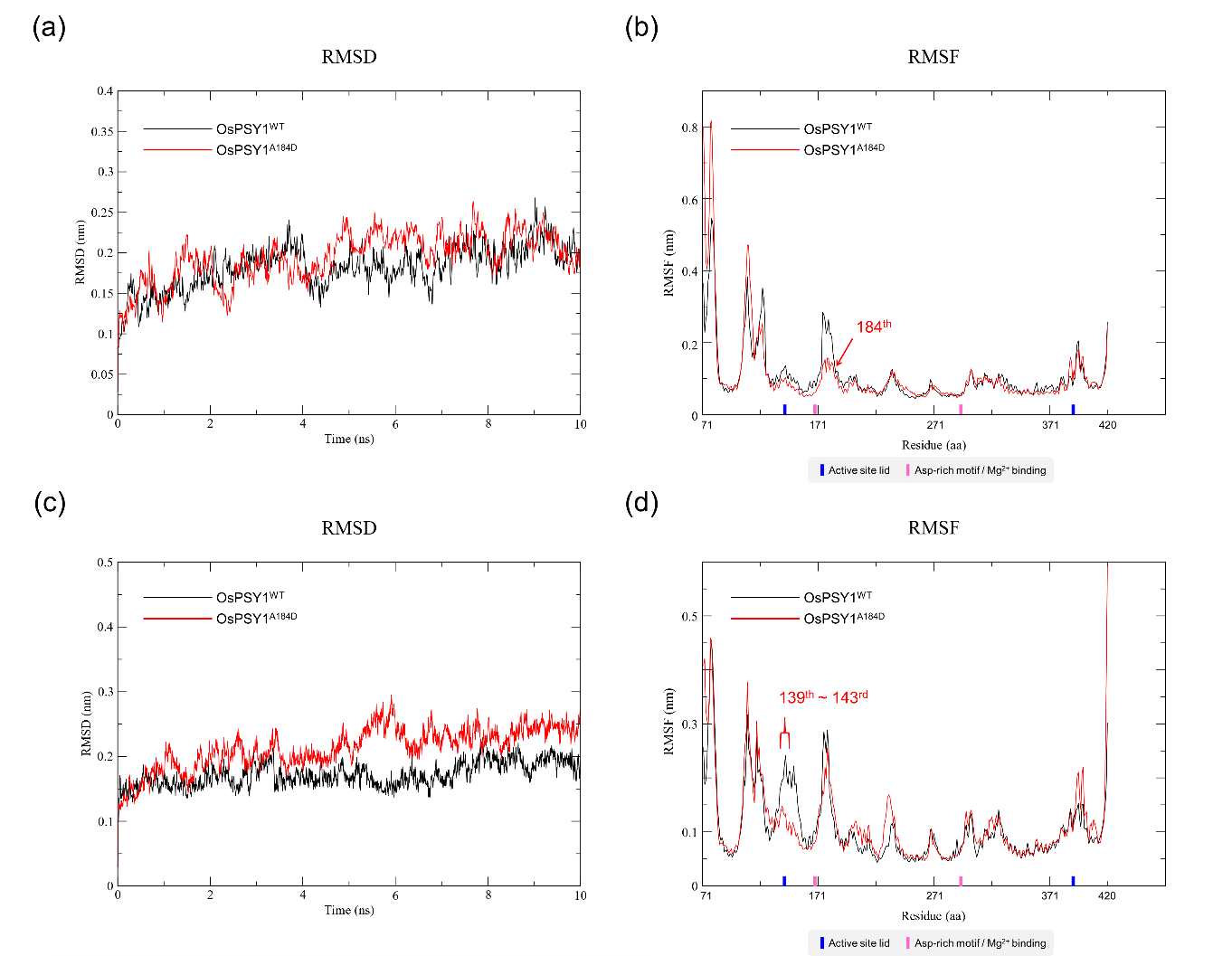
**

**Figure S2. Molecular dynamics analysis of OsPSY1^WT^ and OsPSY1^A184D^ under substrate-free and 2×GGPP-bound conditions.** (a) Protein backbone root-mean-square deviation (RMSD) and (b) root-mean-square fluctuation (RMSF) per residue in the substrate-free simulation. (c) Protein backbone RMSD and (d) RMSF per residue for the 2×GGPP-bound simulation. Residue numbers on the x-axis for RMSF analysis correspond to positions in full-length OsPSY1.

**
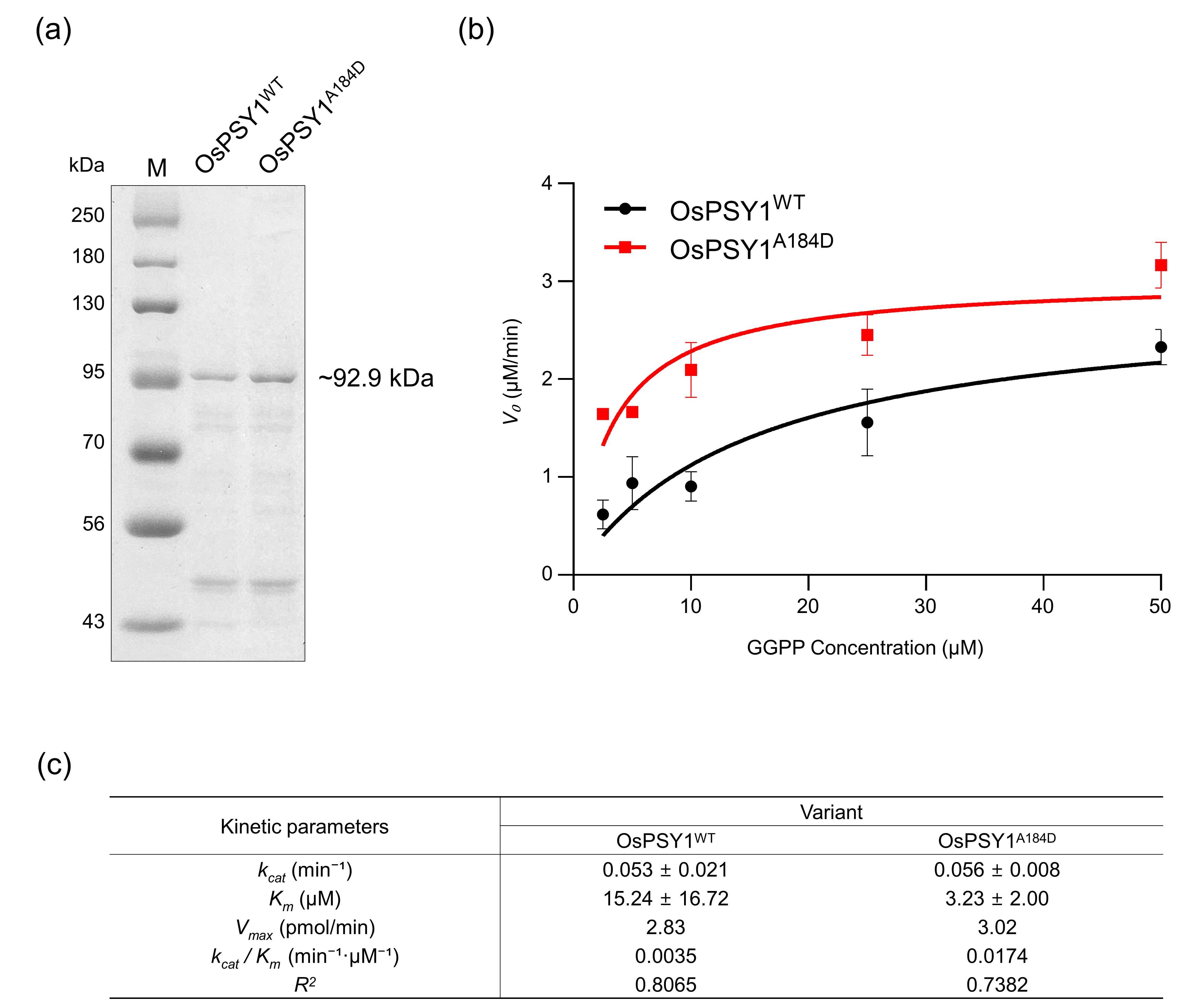
**

**Figure S3. SDS-PAGE and Michaelis–Menten kinetics of OsPSY1 variants.** (a) SDS-PAGE profiles of purified recombinant OsPSY1 variants. Lanes contain OsPSY1^WT^ and OsPSY1^A184D^ fusion proteins (MBP–OsPSY1–His-tag; 43.1‒40.1‒9.7 kDa). M, protein molecular weight marker. (b) Michaelis–Menten plots for two OsPSY1 variants. Initial velocities (*V_0_*) were measured at increasing concentrations of GGPP (2.5, 5, 10, 25, and 50 µM). Data are mean ± SD (*n* = 3). (c) Kinetic parameters of OsPSY1 variants derived from Michaelis–Menten fits of PPi release assays.

**
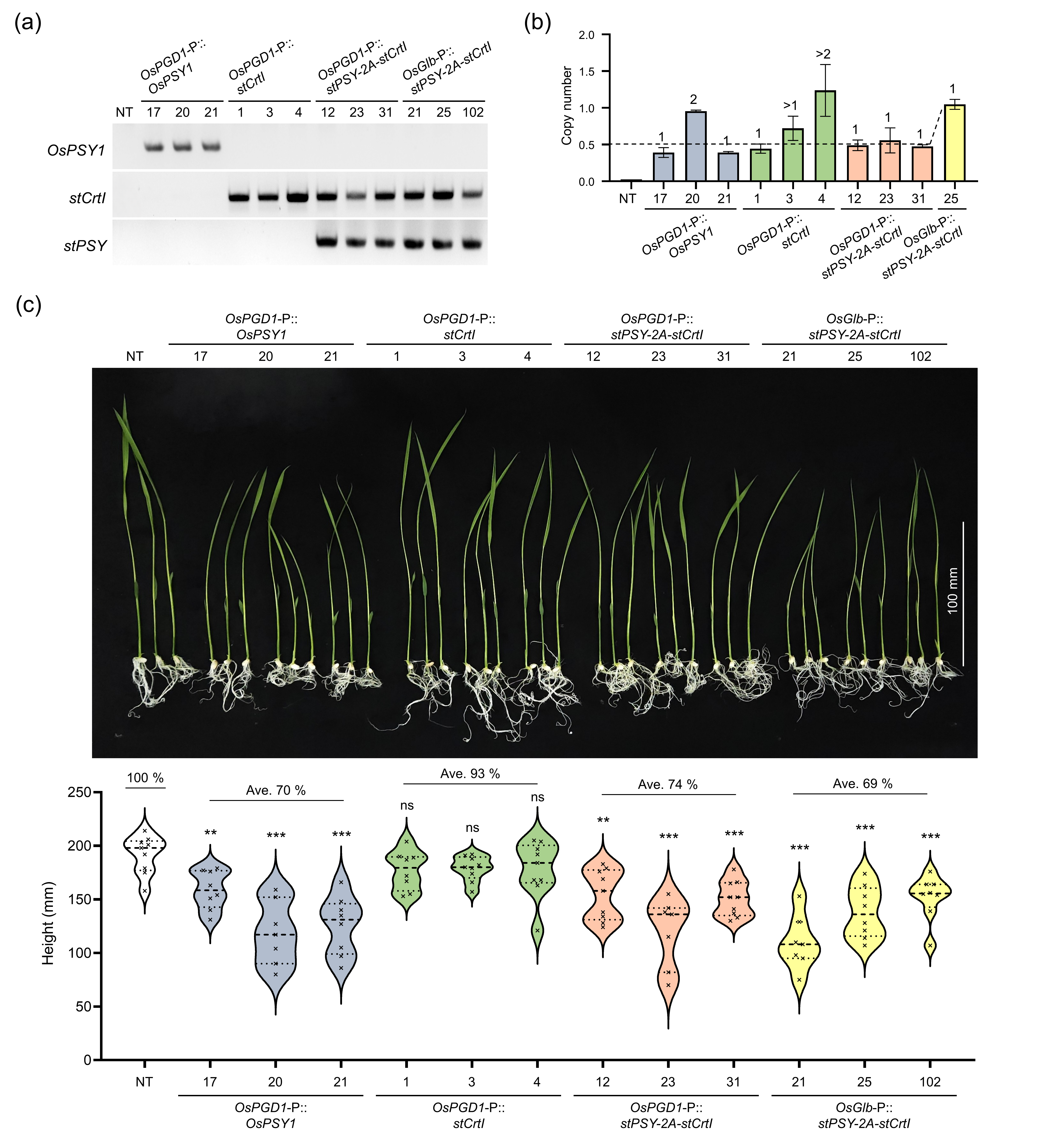
**

**Figure S4. Molecular and phenotypic characterization of transgenic rice seedlings overexpressing carotenoid biosynthetic genes.** (a) Genomic DNA PCR analysis confirming the presence of transgenes in three representative lines per construct (Fig. 1d). (b) TaqMan quantitative PCR analysis for T-DNA copy number determination at heterozygous T_0_ plants, targeting the *Nos* terminator region in *Bar* gene cassette. A single-copy insertion corresponds to a value of 0.5, normalized to the homozygous *OsGlb-*P::*stPSY-2A-stCrtI* (*stPAC*) 25 reference line (value = 1; Jeong et al., 2017). Expected copy numbers are indicated above each bar. (c) Seedling height of NT and transgenic rice lines. Quantitative analysis of seedling height is presented as violin plots (*n* = 10). Asterisks indicate statistical significance determined by Student’s t-test (**P* < 0.05, ***P* < 0.01, and ****P* < 0.001).


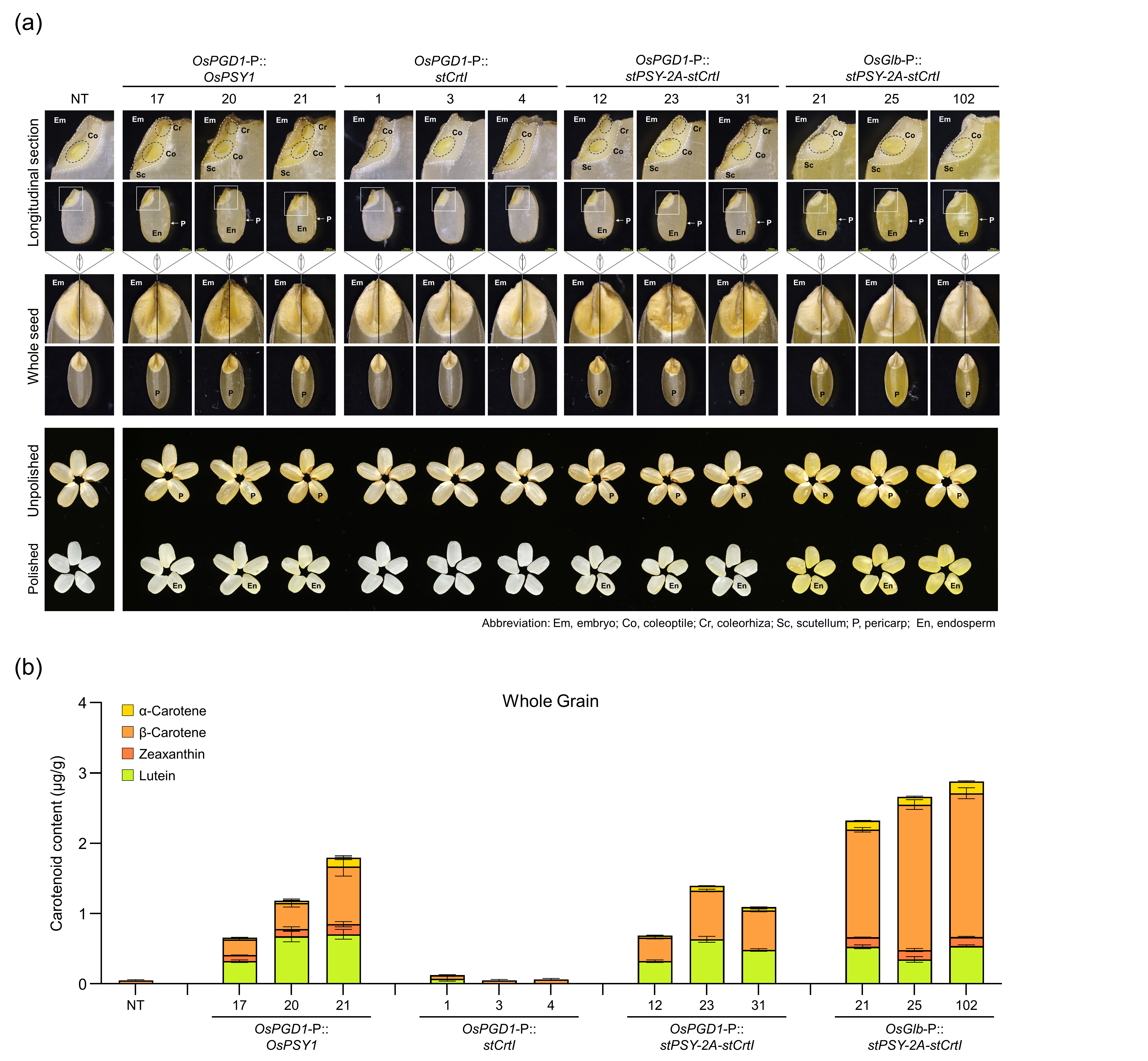


**Figure S5. Phenotypic and carotenoid profiling of transgenic rice seeds overexpressing carotenoid biosynthetic genes.** (a) Seed phenotypes highlighting carotenoid-derived coloration in NT and three representative lines for construct (Fig. 1d). Regions labeled with initials (Em, Co, Cr, Sc, P, and En) exhibit yellow pigmentation, indicating carotenoid accumulation in the corresponding tissues. (b) Carotenoid contents of unpolished whole grains from the corresponding transgenic lines and NT, analyzed by HPLC. Data are mean ± SD (*n* = 3).

**
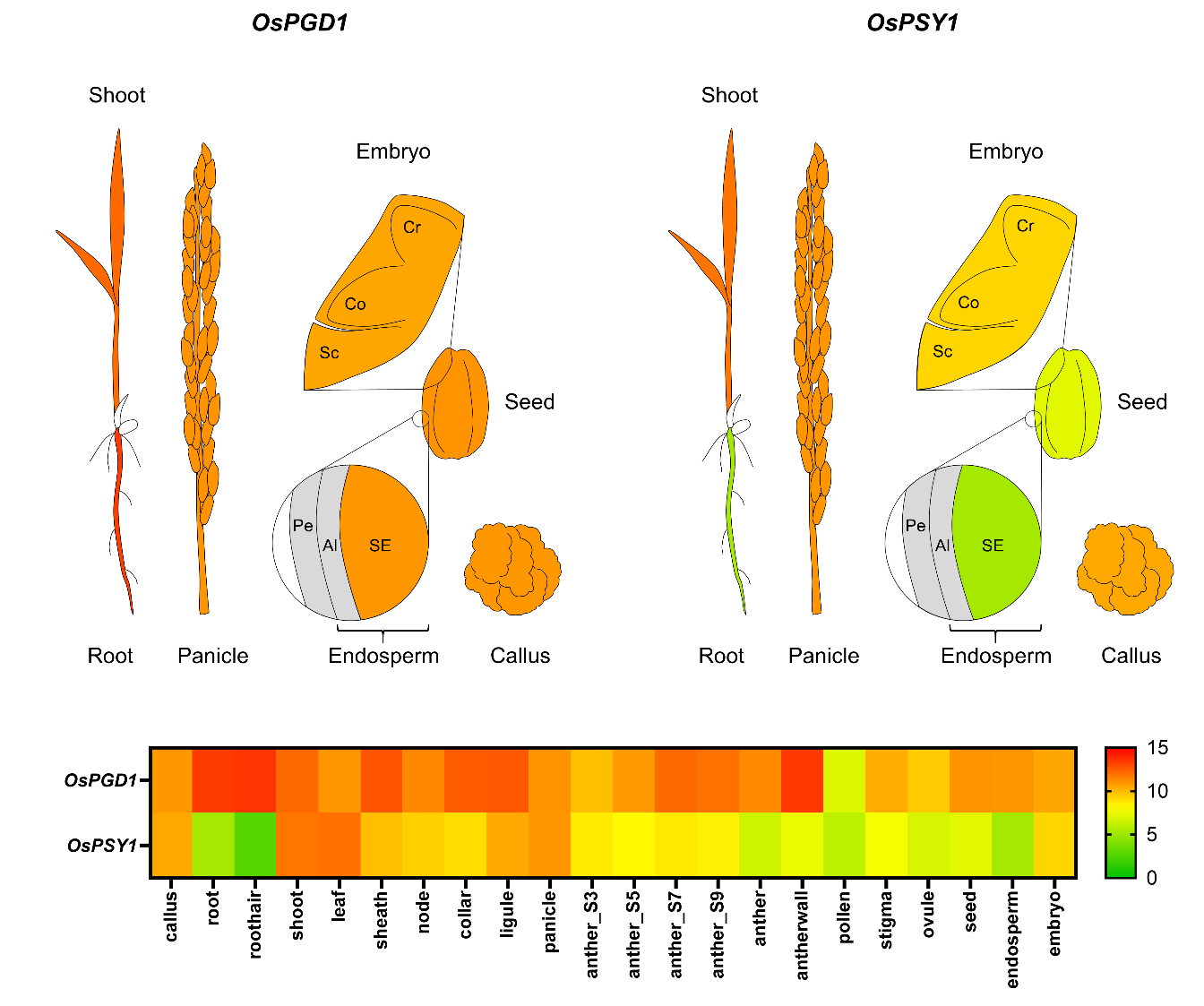
**

**Figure S6. Tissue-specific expression patterns of OsPGD1 and OsPSY1 from public transcriptomic datasets.** Schematics illustrate representative rice tissues with relative expression levels of phosphoglycerate dehydrogenase 1 gene *(OsPGD1*; LOC_Os04g55640 / Os04g0650800; left) and phytoene synthase 1 gene (*OsPSY1*; LOC_Os06g51290 / Os06g0729000; right). The heatmap shows transcript abundance across various tissues and developmental stages, with expression levels indicated the color scale. Data were obtained from the Rice Online Expression Profiles Array Database Version 2 (ROADv2; https://roadv2.khu.ac.kr/). Abbreviation: Co, coleoptile; Cr, coleorhiza; Sc, scutellum; Pe, pericarp; Al, aleurone; SE, starchy endosperm.


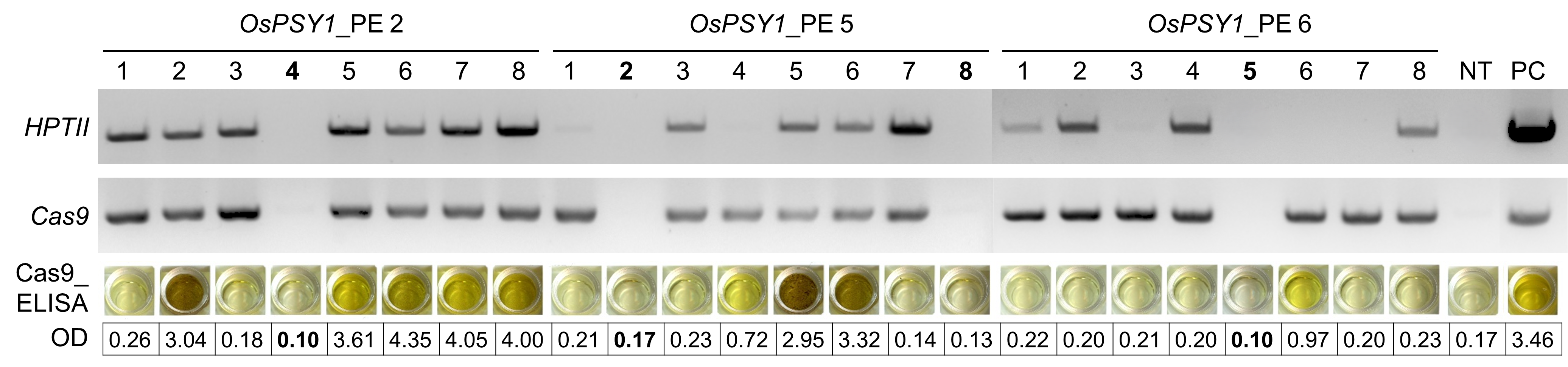


**Figure S7. Transgene-free validation of prime-edited rice lines.** Genomic PCR detecting *HPTII* and *Cas9* genes in three *OsPSY1* prime-edited lines (*OsPSY1*_PE; 2, 5, and 6) and their progeny. Representative images of ELISA-based detection of Cas9 protein. NT, non-transgenic rice plant. PC, positive control with *pPEmax* vector for genomic PCR and purified Cas9 protein for ELISA assay, respectively. OD, optical density at 450 nm. Bold numbers indicate transgene-free lines.

**Table S1. The primer list used in this study.**


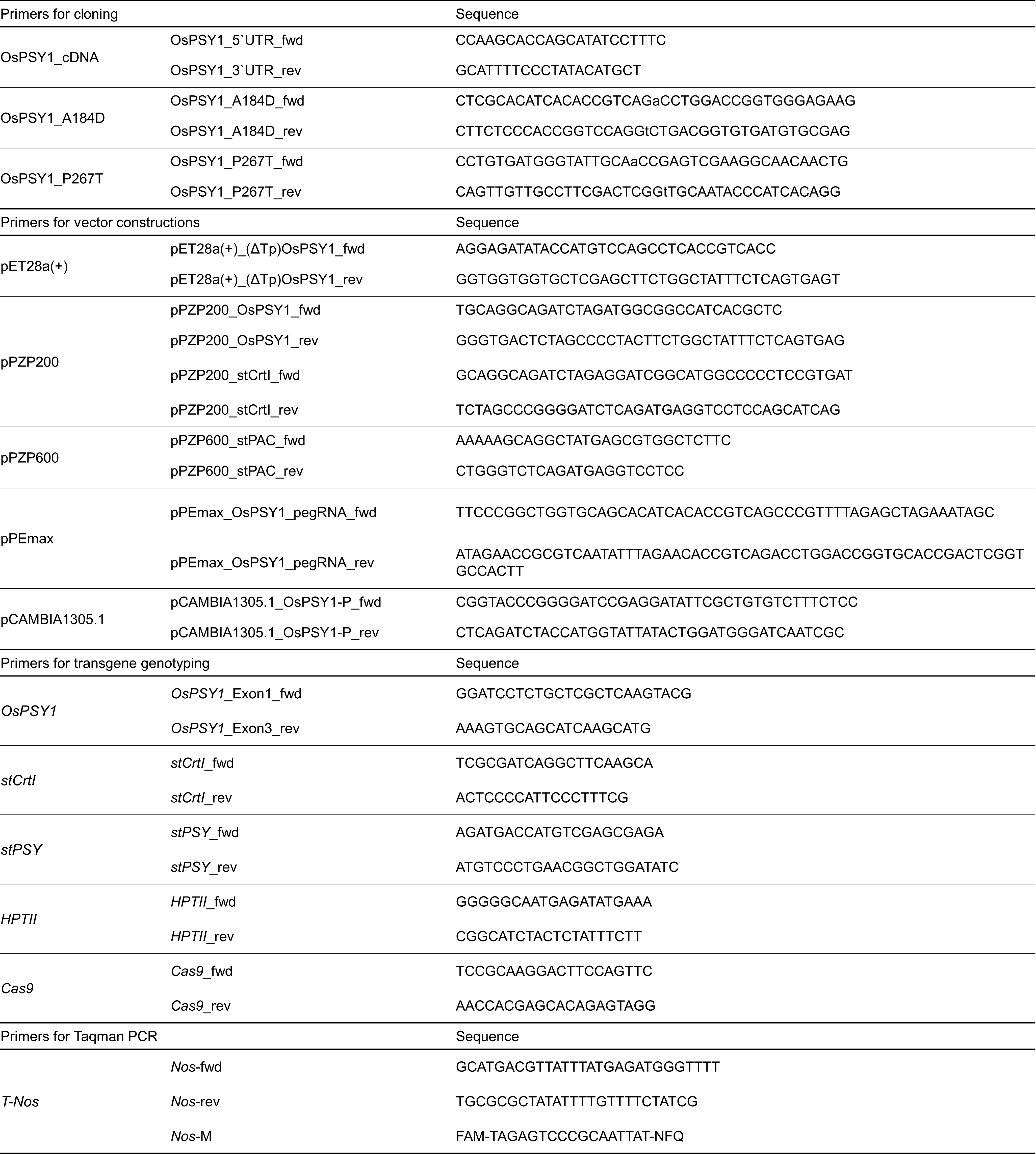


**Table S2.** **Predicted transit peptide and transmembrane regions of OsPSY1.**


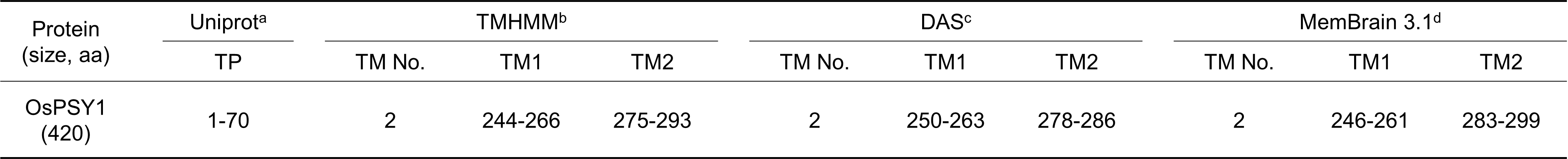


TP, transit peptide; TM, transmembrane; online tools: Uniprot^a,^ (https: //www.uniprot.org/), TMHMM^b^ (https://services.healthtech.dtu.dk/services/TMHMM-2.0/), DAS^c^ (https://tmdas.bioinfo.se/), and MemBrain 3.1^d^ (https://www.csbio.sjtu.edu.cn/bioinf/MemBrain/).


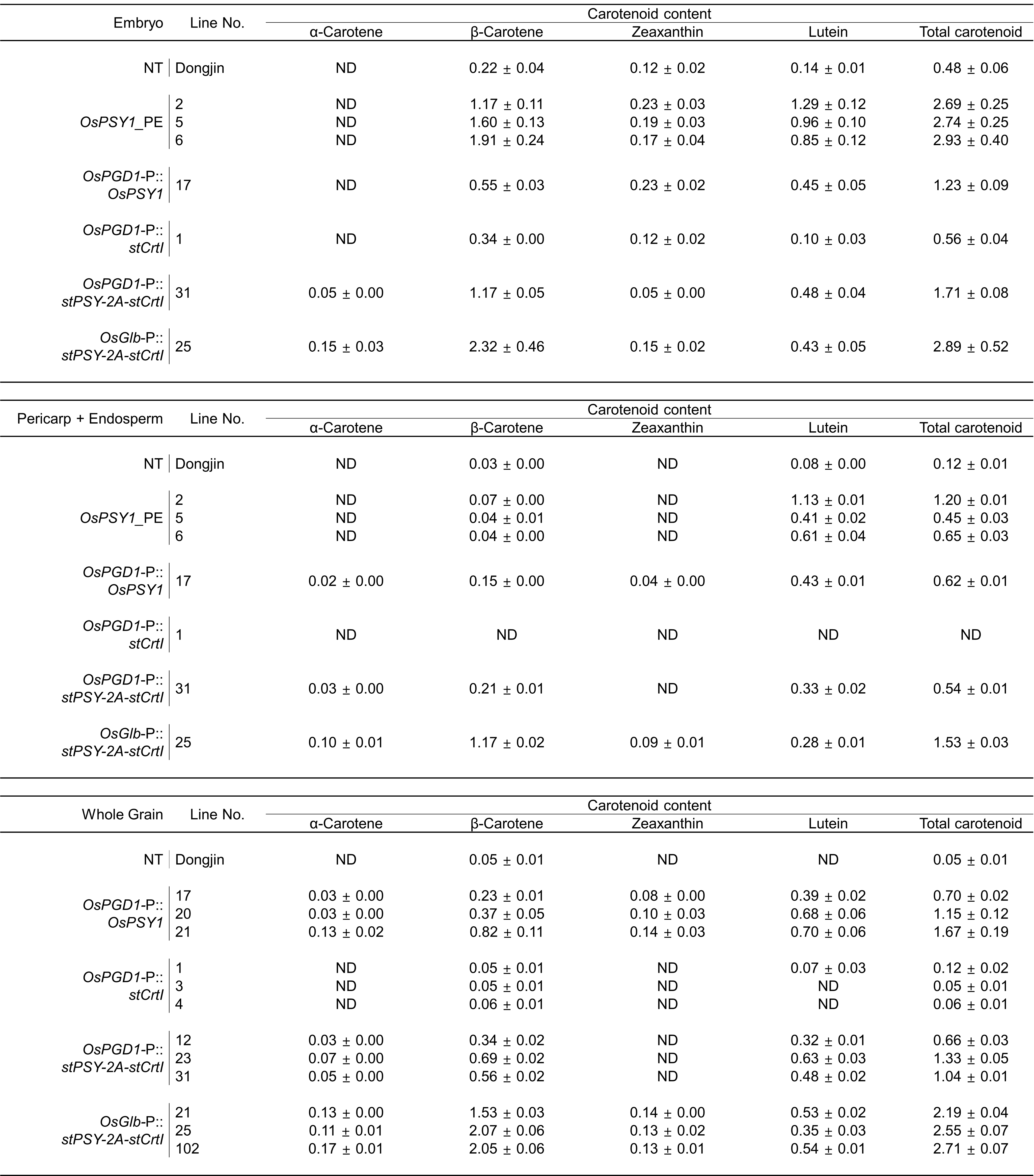
**Table S3.** **Carotenoid contents of rice seeds with embryo, endosperm, and whole grains, respectively.**

Carotenoid contents are expressed as μg/g dry weight.
